# *Panax notoginseng* extract alleviates atopic dermatitis-like skin inflammation associated with suppression of the cGAS–STING pathway

**DOI:** 10.3389/fphar.2026.1859988

**Published:** 2026-07-09

**Authors:** Xu Zhu, Yichun Wang, Zhongyi Chen, Ziyan Zhao, Chunhao Yang, Peng Xu, Liping Qu, Feifei Wang, Fengkun Xiao

**Affiliations:** 1 Yunnan Characteristic Plant Extraction Laboratory Co., Ltd., Kunming, China; 2 Yunnan Botanee Bio-Technology Group Co., Ltd., Kunming, China; 3 Botanee Research Institute, Shanghai Jiyan Biomedical Development Co., Ltd., Shanghai, China

**Keywords:** atopic dermatitis, cGAS–STING pathway, lipid metabolism, *Panax notoginseng* extract, Th2 chemokines

## Abstract

**Background:**

Atopic dermatitis (AD) is a chronic, relapsing inflammatory skin disorder characterized by pruritus and xerosis. Current treatments for AD primarily involve glucocorticoids and immunosuppressive agents. However, long-term administration of these treatments may lead to significant adverse effects. Therefore, there is an urgent need for effective therapeutic alternatives with improved safety profiles. *Panax notoginseng* (Burkill) F. H. Chen is renowned for its diverse pharmacological properties and shows promise in the treatment of skin diseases.

**Purpose:**

This study investigated the effects of *P. notoginseng* extract (PNE) on AD and its underlying mechanisms.

**Methods:**

We analyzed the chemical composition of PNE using high-performance liquid chromatography (HPLC). The anti-AD effects of PNE were evaluated in both an AD-like mouse model induced by calcipotriol (MC903) and a human keratinocyte (HaCaT) cell model stimulated with tumor necrosis factor-alpha and interferon-gamma (TNF-α/IFN-γ). Furthermore, metabolomics analysis of mouse skin tissue was conducted to elucidate the underlying metabolic mechanisms. To investigate the associated signaling pathways, quantitative real-time PCR (qPCR) and Western blotting were performed.

**Results:**

In MC903-induced mice, PNE exhibited potent anti-AD effects, including attenuation of AD-like skin lesions and suppression of Th2 cytokines (*Il-4*, *Il-13*, *Il-31*) and chemokines (*Ccl5*, *Ccl17*, *Ccl22*). In TNF-α/IFN-γ-stimulated HaCaT cells, PNE dose-dependently inhibited the expression of Th2-associated chemokines (*CCL5*, *CCL17*, *CCL22*). Moreover, PNE suppressed the mRNA expression of *cGAS*, *STING*, *IRF3*, *IRF7*, and *TBK1*, as well as the protein levels of cGAS and STING, both *in vitro* and *in vivo*. Metabolic profiling of mouse skin tissues revealed that PNE significantly modulates the metabolism of phosphatidylcholine, phosphatidylethanolamine, and arachidonic acid. HPLC analysis showed that ginsenoside Rg_1_, notoginsenoside R_2_(*S*), ginsenoside Rb_1_, and ginsenoside Rd were the main components of PNE. Notoginsenoside R_2_(*S*) and ginsenoside Rd dose-dependently inhibited the mRNA expression of *CCL5*, *CCL17*, *CCL22*, *cGAS*, *STING*, *IRF3*, *IRF7*, and *TBK1*.

**Conclusion:**

PNE exerts anti-AD effects by attenuating Th2-mediated inflammatory responses via modulation of the cGAS–STING pathway. These findings suggest that PNE has the potential to serve as a natural therapeutic option for AD.

## 1 Introduction

Atopic dermatitis (AD) is a chronic, recurrent, and inflammatory skin disorder. The global prevalence of this condition has been steadily increasing, posing a growing challenge to public health systems (Nasr and Ghonaim, 2014). Affecting approximately one-fifth of children and one-tenth of adults worldwide, AD places considerable strain on healthcare resources across countries (Li et al., 2025). Patients diagnosed with AD frequently experience comorbid allergic disorders—including asthma, allergic rhinitis with conjunctivitis, and food hypersensitivity—making therapeutic management more complex (Silverberg, 2019). First-line treatments typically involve topical corticosteroids and calcineurin inhibitors. However, prolonged use carries notable safety concerns, including cutaneous atrophy, systemic glucocorticoid-related adverse effects, and potential nephrotoxicity (Bieber, 2022).

AD arises from a multifaceted interplay among inherited susceptibility, external environmental factors, and dysregulated immune responses (Boguniewicz and Leung, 2011; Schmuth et al., 2024). AD is characterized primarily by a Th2-polarized immune response. The cytokines IL-4, IL-13, and IL-31 contribute to persistent skin inflammation and pruritus by disrupting keratinocyte homeostasis and promoting the recruitment of proinflammatory immune cells (Brandt and Sivaprasad, 2011). Thymic stromal lymphopoietin (TSLP) is a pivotal cytokine that drives and sustains Th2-type immune responses, thereby perpetuating cutaneous inflammatory processes (Schmuth et al., 2024). With the progression of AD, the immune response becomes increasingly complex, involving both Th1 and Th2 lymphocyte subsets, as well as elevated levels of tumor necrosis factor-alpha (TNF-α) and interferon-gamma (IFN-γ) (Tsoi et al., 2020). Upon activation by diverse stimuli, keratinocytes exacerbate AD pathology by secreting chemokines (CCL5 (CC chemokine ligand 5), CCL17, and CCL22) that recruit Th2 cells, thereby promoting their infiltration into lesional skin (Das et al., 2022). Therefore, an integrated therapeutic strategy targeting both keratinocytes and components of the immune system is essential for the successful management of AD.

Calcipotriol (also known as MC903), a synthetic vitamin D_3_ derivative, is used to induce an AD mouse model that is currently regarded as closely recapitulating the clinical features of human AD (Hoshino et al., 2025). It directly triggers Th2-type immune responses, which are associated with the pathogenesis of human AD (Huang, D. et al., 2025). This model recapitulates multiple core pathological features of human AD, including epidermal hyperplasia, impaired skin barrier function, severe pruritus, and elevated serum total IgE levels (Huang, R. et al., 2025). Consequently, it has been extensively used in research on AD pathogenesis and in preclinical drug screening. For instance, studies have shown that the traditional Chinese medicine ointment Chushi Zhiyang Ruangao effectively alleviates dermatitis in this murine model (Zeng et al., 2024); likewise, anti-pruritic research on sophoramine has also utilized this model (Lou et al., 2026). Moreover, this model has demonstrated that a novel nuclear transport inhibitor can treat AD by suppressing TSLP expression (Liu et al., 2022).

*Panax notoginseng* (PN) (Burkill) F. H. Chen is a traditional medicinal plant native to southwestern China. It is widely used in clinical formulations, dietary supplements, and cosmetic products worldwide (Liu, H. et al., 2020). PN has attracted increasing scientific interest due to its pharmacological effects in skin whitening and reduction of hyperpigmentation (Chen et al., 2025). Saponins extracted from PN exhibit strong protective effects against inflammatory injury to the skin barrier (Xie et al., 2025). Notoginsenoside R1, a pharmacologically active saponin isolated from PN, exhibits protective effects against UVB-induced skin damage (Liu et al., 2026). Experimental evidence has confirmed that saponins extracted from PN exhibit potent anti-inflammatory activity (Wang et al., 2025). Recent studies have revealed the remarkable immunomodulatory effects of PN (Yang et al., 2024). However, its application in the treatment of AD requires further exploration. Herein, an MC903-induced mouse model, combined with inflammatory cell models, was employed to comprehensively assess the effect of *P. notoginseng* extract (PNE) on AD and to elucidate the underlying mechanisms.

## 2 Materials and methods

### 2.1 Reagents and chemicals

The HaCaT cell line was obtained from the Cell Bank of the Chinese Academy of Sciences (species: human, CVCL number: 0038, CN: SCSP-5091). Dulbecco’s Modified Eagle Medium (DMEM) (CN: 10566016), Trypsin solution (0.25%) (CN: R001100), and Fetal Bovine Serum (FBS) (CN: 16000069) were obtained by Gibco (Thermo Fisher Scientific, Waltham, MA, USA). Sigma-Aldrich China (Shanghai, China) provided the following reagents: phosphate-buffered saline (PBS) (CN: P2272), phosphoric acid (CN: 49685), HPLC-grade acetonitrile (CN: 34851), and methanol (CN: 34860). RIPA lysis buffer (CN: R0010), phenylmethanesulfonyl fluoride (PMSF) (CN: P0100), human CCL5 (CN: SEKH-0061), CCL17 (CN: SEKH-0315), and CCL22 (CN: SEKH-0239) ELISA kits were purchased from Beijing Solepol Technology Co., Ltd. (Beijing, China). The following compounds were all purchased from the China Institute for Food and Drug Control: ginsenoside Rg_1_ (G-Rg_1_, batch number: 110703-202235, purity: 98.5%), ginsenoside Rb_1_ (G-Rb_1_, batch number: 110704-202230, purity: 95.1%), ginsenoside Rd (G-Rd, batch number: JB238591, purity: 98%), and notoginsenoside R_2_(*S*) (NG-R_2_(*S*), batch number: P04J12S136477, purity: 95%). MC903 was obtained from Shanghai McLean Biochemical Technology Co., Ltd. (Shanghai, China). Dexamethasone was obtained from MedChemExpress (NJ, USA). Molecular biology reagents were used in this study, including primers supplied by BGI Genomics Co., Ltd. (Shenzhen, China) and the PrimeScript™ RT Reagent Kit for reverse transcription provided by Takara Bio. The dried roots of PN were obtained from Yunnan Yaotong Traditional Chinese Medicine Resources Development Co., Ltd. (Yunnan, China) in November 2024. The botanical identity was verified by Professor Li Guodong from Yunnan University of Traditional Chinese Medicine. The verified voucher specimen (YNWS20241106-1) is archived at Yunnan Characteristic Plant Extraction Laboratory.

### 2.2 Preparation of PNE

The crushed plant material of the PN root (100 g) was subjected to reflux extraction using an 85% (v/v) ethanol-water solution at 100 °C, with each extraction cycle lasting 2 hours. Subsequently, the pooled filtrates were loaded onto a D101 macroporous adsorption resin column and eluted stepwise with deionized water (4 bed volumes, 4 BV), 30% ethanol (5 BV), 50% ethanol (7 BV), and 60% ethanol (7 BV). The PNE was obtained by concentrating the fraction collected from the 60% ethanol elution under reduced pressure and then drying it under vacuum. The extraction yield of PNE is 7.68%, and batch-to-batch consistency data for PNE are provided in Supplementary Table 1.

### 2.3 Component analysis of PNE

The study employed an Agilent 1290 Infinity II HPLC system equipped with a photodiode array detector for analysis. Sample preparation involved the precise weighing of PNE specimens, followed by dissolution in HPLC-grade methanol. For quantitative analysis, a mixed standard solution containing G-Rg_1_, NG-R_2_(*S*), G-Rb_1_, and G-Rd (1 mg/mL each) was prepared. Both the sample and reference solutions were subjected to identical chromatographic conditions for peak identification and method validation. Chromatographic separation was performed on an Agilent ZORBAX SB-C18 column (4.6 mm × 250 mm, 5 μm) operated at a constant temperature of 25 °C. The elution system employed a binary gradient composed of ultrapure water (mobile phase A) and acetonitrile (mobile phase B). The elution program was as follows: from 0 to 30 min, the ratio of A to B was maintained at 81:19 (v/v); from 30 to 65 min, the ratio of A to B was adjusted to 64:36 (v/v); from 65 to 70 min, the ratio of A to B was adjusted to 60:40 (v/v); from 70 to 80 min, the ratio of A to B was maintained at 60:40 (v/v); from 80 to 81 min, the ratio of A to B was adjusted to 0:100 (v/v); from 81 to 85 min, the ratio of A to B was maintained at 0:100 (v/v). The flow rate was 1 mL/min. UV detection at 203 nm was used for analysis, and a 5 μL sample was injected into the system.

### 2.4 Animal experiments

The experimental subjects were male BALB/c mice, 7–8 weeks old and weighing 20–25 g, purchased from Beijing Vital River Laboratory Animal Technology Co., Ltd. The animal quality certification number is 110011241110592062. The experiments were conducted in a climate-controlled environment with a temperature maintained between 20 °C and 26 °C and relative humidity ranging from 35% to 75%, under a standardized 12-h light/12-h dark photoperiod. The animal experiment was conducted by Hefei Zhongkepuruisheng Biomedical Technology Co., Ltd. The experimental protocol was formally reviewed and approved by the Laboratory Animal Welfare and Ethics Committee of Hefei Zhongkepuruisheng Biomedical Technology Co., Ltd. (Approval No.: IACUC-20241101).

AD-like skin lesions were induced in BALB/c mice using MC903, as previously described (Lee et al., 2023). Following a 7-day adaptation period, the mice were randomly assigned to six experimental groups using a random number table (n = 6 per group): (1) negative control group (CON: treated with absolute ethanol alone), (2) MC903-treated group (Model), (3) MC903-treated + PNE-low (4.5 mg/kg) group, (4) MC903-treated + PNE-middle (9 mg/kg) group, (5) MC903-treated + PNE-high (18 mg/kg) group, and (6) MC903-treated + dexamethasone (DEX) group. During the experimental procedure, the dorsal coat of each mouse was initially clipped with an electric clipper, and any residual hair was then removed using a depilatory agent. MC903, PNE, and DEX were all prepared using absolute ethanol. AD was experimentally induced in BALB/c mice by daily topical application of 40 μL of absolute ethanol containing 90 μM MC903 to the dorsal skin surface. Various doses of PNE (4.5, 9, and 18 mg/kg) were topically applied to the same areas once daily for 13 consecutive days to assess its effect on mitigating MC903-induced AD-like manifestations. In the CON group, absolute ethanol alone was applied to the same dorsal skin areas. PNE was applied to back in groups 4, 5, and 6. The positive control group was topically treated with DEX at 1.2 mg/kg (days 0–5) and 0.4 mg/kg (days 5–13). Body weight of the mice was recorded daily throughout the entire experimental period. On day 14, blood samples were obtained via retro-orbital bleeding, followed by centrifugation at 2000 g for 15 min to separate serum, which was then aliquoted and preserved at −80 °C pending further assays. Concurrently, dorsal skin specimens were harvested and stored at −80 °C for downstream analyses. An overview of the experimental timeline and procedures is shown in Figure 2A. The clinical manifestations were assessed in line with the following criteria: erythema, edema/papules, dryness/desquamation, and lichenification. Subsequently, these manifestations were categorized into four grades: none, mild, moderate, and severe, with scores of 0, 1, 2, and three assigned respectively. The skin on the animals’ backs was scored on a daily basis. The specific assessment criteria are presented in Supplementary Table 2.

### 2.5 Histological examination

To assess the effect of PNE, histopathological analysis was performed on dorsal skin specimens. Skin tissues were harvested from each mouse, fixed in 10% neutral-buffered formalin, processed for paraffin embedding, and sectioned at a thickness of 4 μm. Epidermal thickening was then evaluated using hematoxylin and eosin (H&E) staining.

### 2.6 Measurement of IgE levels and Th2 cytokines

Serum IgE concentrations, along with tissue levels of IL-4, IL-13, IL-31, and TSLP in skin samples, were quantified using commercially available enzyme-linked immunosorbent assay (ELISA) kits (Solarbio, Beijing, China) according to the manufacturer’s instructions.

### 2.7 Cell experiments

HaCaT cells were cultured in DMEM with 10% FBS and 1% penicillin–streptomycin at 37 °C and 5% CO_2_. The cytotoxic effects of PNE on HaCaT cells were evaluated. Cells were seeded into 96-well microplates at a density of 1 × 10^4^ cells per well. After 24 h for attachment and growth to approximately 80% confluence, the cells were treated with various concentrations of PNE for another 24 h. Next, add 10 μL CCK-8 reagent to each well and incubate at 37 °C for 1 h. Measure absorbance at 450 nm using a microplate reader.

HaCaT cells were seeded into 6-well culture plates at a density of 4 × 10^5^ cells per well. After 24 h incubation (37 °C, 5% CO_2_), the medium was replaced with fresh DMEM containing PNE at concentrations of 60 μg/mL, 120 μg/mL, and 240 μg/mL. Cells were then treated with PNE for 1 h, followed by TNF-α (10 ng/mL) and IFN-γ (10 ng/mL) for 23 h. Finally, cells were harvested for RNA or protein extraction.

HaCaT cells were seeded into 6-well culture plates at a density of 4 × 105 cells per well. After 24 h incubation (37 °C, 5% CO2), the medium was replaced with fresh DMEM. Cells were pre-incubated with 2 μM STING agonist SR-717 (CN: GC61293, GLPBIO) for 2 h. Subsequently, Cells were incubated with PNE for 1 h, then stimulated with TNF-α (10 ng/mL) and IFN-γ (10 ng/mL) for 23 h. After treatment, cells were harvested for RNA extraction, and supernatants were collected for ELISA.

HaCaT cells were seeded into 6-well culture plates at a density of 4 × 105 cells per well. After 24 h incubation (37 °C, 5% CO2), the medium was replaced with fresh DMEM. Cells were pre-incubated with 1 μM STING inhibitor H151 (CN: GC34610, GLPBIO) for 6 h. Subsequently, Cells were incubated with PNE for 1 h, then stimulated with TNF-α (10 ng/mL) and IFN-γ (10 ng/mL) for 23 h. After treatment, cells were harvested for RNA extraction, and supernatants were collected for ELISA.

### 2.8 RNA extraction and qPCR

Total RNA was extracted from HaCaT cells using the GeneJET™ RNA Purification Kit (CN: K0732, Thermo Fisher Scientific, USA) and from mouse skin tissue using TRIzol® reagent. cDNA was synthesized via reverse transcription with PrimeScript™ RT Master Mix (CN: RR036B; Takara Bio Inc., Japan). qPCR was performed using FastStart Essential DNA Green Master Mix (CN: 06402712001, Roche Diagnostics GmbH, Germany). Primer sequences are listed in Supplementary Tables 3, 4. Relative mRNA expression was calculated using the 2^−ΔΔCt^ method, normalized to GAPDH.

### 2.9 Western blot analysis

HaCaT cells and mouse skin tissues were lysed in RIPA buffer with PMSF and phosphatase inhibitors (100:1:1). Lysates were centrifuged at 12,000 × g for 10 min at 4 °C; supernatants were collected and protein concentration measured using a BCA assay kit (CN: P0012, Beyotime Biotechnology, Shanghai, China). Proteins were separated by SDS-PAGE on commercial gradient polyacrylamide gels and transferred to nitrocellulose membranes. Membranes were blocked for 1 h at 25 °C with TBST (0.1% Tween-20) containing 5% (w/v) BSA. Primary antibodies—cGAS (mouse, 1:1000, CN: A0674, RRID: AB_2757326, ABclonal Biotechnology, Wuhan, China), STING (rabbit, 1:5000, CN: A1346, RRID: AB_2760320, ABclonal Biotechnology, Wuhan, China), IRF3 (rabbit, 1:1000, CN: A27272, ABclonal Biotechnology, Wuhan, China), TBK1 (rabbit, 1:1000, CN: A3458, ABclonal Biotechnology, Wuhan, China), Phospho-IRF3 (Ser396) (rabbit, 1:1000, CN: 29047, Cell signaling technology, USA), Phospho-TBK1 (Ser172) (rabbit, 1:1000, CN:5483, Cell signaling technology, USA), and β-actin (mouse, 1:10000, CN: AC002, RRID: AB_2736879, ABclonal Biotechnology, Wuhan, China—were applied overnight at 4 °C. After three TBST washes, membranes were incubated for 1 h at 25 °C with HRP-conjugated secondary antibodies: goat anti-rabbit IgG (1:10000, CN: A-11034, RRID: AB_2576217, Thermo Fisher Scientific, Waltham, MA, USA) or goat anti-mouse IgG (1:10000, CN: A-21235, RRID: AB_2535804, Thermo Fisher Scientific, Waltham, MA, USA). Protein bands were visualized using an ultra-sensitive ECL substrate (CN: P0018M, Beyotime Biotechnology, Shanghai, China) and imaged on a Tanon-5200 system. Band intensities were quantified using ImageJ and normalized to β-actin.

### 2.10 Metabolomics analysis

Metabolite profiling is a powerful tool for elucidating the molecular mechanisms underlying diverse disease states. To elucidate how PNE modulates MC903-induced AD, we performed untargeted metabolomics analysis to identify candidate biomarkers. Samples were analyzed by LC–MS/MS at OE Biotech Co., Ltd. (Shanghai, China). Each group of test samples consists of six biological specimens. The quality control (QC) sample is prepared by mixing equal volumes of the extraction solutions from all samples. The loading volume is identical to the sample volume. A QC sample is inserted after every four test samples to assess the repeatability of the entire analytical process. Ion source: HESI. The mass spectrometry signals of the samples were acquired using both positive- and negative-ion scanning modes. Compound identification was based on multiple orthogonal criteria, including retention time (RT), accurate mass, MS/MS fragment spectra, and isotope distribution. Identification and annotation were conducted using the Human Metabolome Database (HMDB), Lipid maps, METLIN database, and the LuMet-Animal3.0. LuMet-Animal3.0 is an LC-MS/MS database established by OE Biotech Co., Ltd. The resulting data matrix was processed using Mass Profiler Professional software (Agilent Technologies, Santa Clara, CA, USA) and subjected to multivariate statistical analysis—orthogonal partial least squares discriminant analysis (OPLS-DA)—using SIMCA-P+ 13.0 (Umetrics, Kinnelon, NJ, USA). Screening criteria for differential metabolites: Statistical parameters calculated based on all metabolites in the original data matrix, including *p*-value <0.05, Fold Change ≥2.0 or Fold Change ≤0.5, and VIP ≥1. Multiple testing correction was performed using false discovery rate (FDR) method.

### 2.11 Molecular docking of STING

To perform AutoDock simulations, the BIOVIA Discovery Studio 2019 software was utilized. Initially, the three-dimensional crystal structure of the STING protein (PDB code: 6O8C) was downloaded from the Protein Data Bank. Meanwhile, the molecular structures of G-Rg1, NG-R_2_(*S*), G-Rb1, and G-Rd were acquired from PubChem. Subsequently, energy minimization was performed on the ligand molecules, and molecular docking was conducted using the CD DOCKER. Docking Parameters is Top Hits: 10, Random Conformations: 10, Orientations to Refine: 10, Simulated Annealing: True. Discovery Studio 2019 was employed to conduct visual analysis on the molecular docking results, and two-dimensional and three-dimensional interaction maps were constructed. The binding affinity values of each ligand to the target protein were also obtained through calculation. When calculating the binding energy, the MM-PBSA mode was adopted to eliminate the influence of solvent during the calculation process.

### 2.12 Statistical analysis

All results are presented as the mean ± standard deviation (SD), and statistical analyses were performed using GraphPad Prism version 8 (GraphPad Software, La Jolla, CA, USA). To evaluate intergroup differences, one-way analysis of variance (ANOVA) was conducted, followed by Tukey’s *post hoc* test for pairwise comparisons. Statistical significance was set as *p* < 0.05.

## 3 Results

### 3.1 Major components of PNE

**FIGURE 1:**
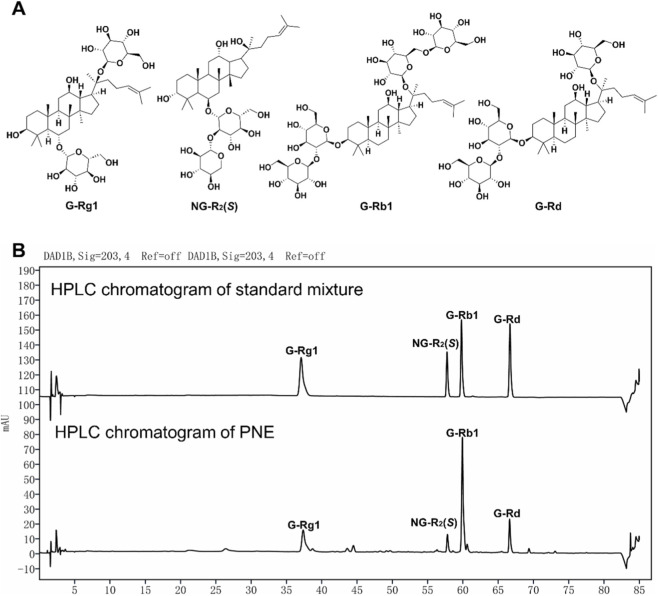
Identification of metabolites in *Panax notoginseng* extract (PNE). **(A)** Chemical structures of G-Rg_1_, NG-R_2_(*S*), G-Rb_1_, and G-Rd. **(B)** HPLC chromatograms of the standard mixture and PNE.

HPLC was used to analyze the chemical composition of PNE. As illustrated in the representative chromatogram (Figure 1B), the identities of four metabolites (G-Rg_1_, NG-R_2_(*S*), G-Rb_1_, and G-Rd) were verified by matching their RT and UV spectral characteristics with those of certified reference standards. To enable quantitative determination, calibration curves for these four analytes were constructed via linear regression of peak area against corresponding concentrations. The calibration curve for G-Rg_1_ exhibited excellent linearity with the equation y = 345.93x + 7.4867 (*R* ^2^ = 0.9985), for NG-R_2_(*S*) by y = 384.88x + 8.3783 (*R* ^2^ = 0.9972), for G-Rb_1_ by y = 303.57x – 13.516 (*R* ^2^ = 0.9979), and G-Rd y = 408x – 1.8431 (*R* ^2^ = 0.9975). The analysis confirmed the respective contents of G-Rg_1_, NG-R_2_(*S*), G-Rb_1_, and G-Rd as 14.5%, 4.24%, 41.61% and 8.13% in PNE.

### 3.2 PNE ameliorates MC903-induced AD-like symptoms in mice

**FIGURE 2:**
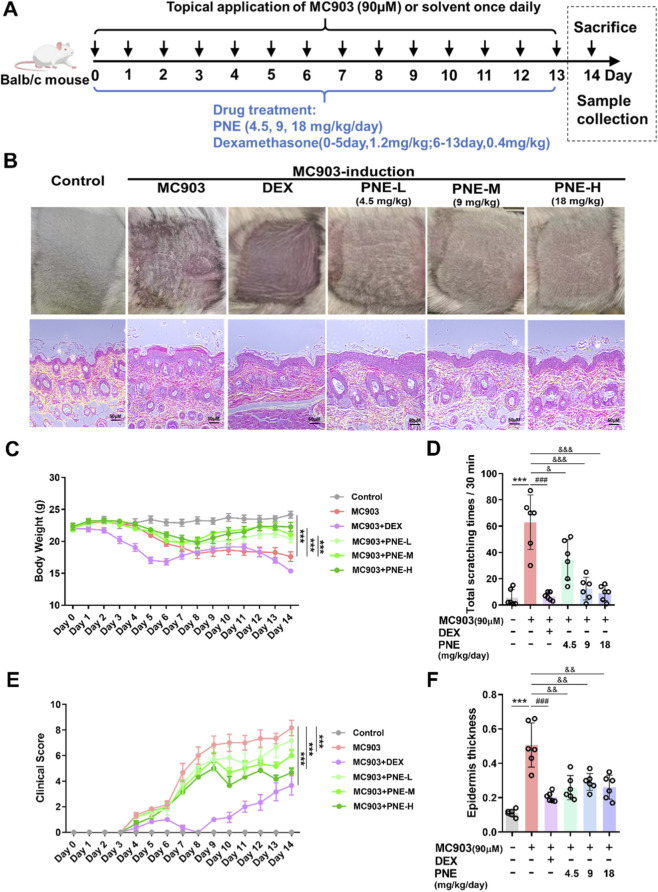
Effects of PNE on AD-like symptoms in MC903-induced mice. **(A)** Schematic illustration of the experimental procedure. **(B)** Representative photographs showing the dorsal skin of mice from various experimental groups, along with corresponding hematoxylin and eosin (H&E)-stained histological sections of skin tissue. **(C)** Changes in body weight. **(D)** Total scratching time. **(E)** Clinical scores. **(F)** Epidermal thickness was assessed using hematoxylin and eosin (H&E)-stained skin tissue sections. Values are expressed as mean ± SD (n = 6). Statistical significance was assessed as follows: comparisons between the MC903 and control group are indicated by *P < 0.05, **P < 0.01, ***P < 0.001; between the DEX and MC903 group by #*P* < 0.05, ##*P* < 0.01, ###*P* < 0.001; and between the PNE and MC903 group by &*P* < 0.05, &&*P* < 0.01, &&&*P* < 0.001.

To explore the potential effects of PNE in a mouse model of AD, BALB/c mice were subjected to dorsal skin inflammation induced by MC903 (Figure 2). Compared with the control group, MC903-treated mice developed marked epidermal hyperplasia, erythema, and edema—hallmarks consistent with AD pathology. Importantly, topical application of either PNE or the DEX (positive drug) substantially ameliorated these clinical manifestations, as quantitatively reflected in Figure 2B. Consistently, hematoxylin and eosin (H&E) staining revealed that PNE treatment significantly attenuated epidermal thickening in AD-affected mice (Figure 2F). Compared to the control group, MC903-treated mice exhibited a significant decrease in body weight. Notably, topical treatment with medium- and high-concentration PNE (9 and 18 mg/kg) improved body weight in these mice. Mice administered MC903 exhibited a significantly higher clinical score than those in the untreated control group. In both the DEX and PNE treatment groups, the clinical score was alleviated, with statistically significant decreases observed in the medium- and high-dose PNE (9 and 18 mg/kg) groups (Figure 2E). The total scratching times per 30 min was markedly elevated in MC903-induced mice than in the control group, whereas DEX and PNE significantly reduced the total scratching times per 30 min on the final day of the experiment (Figure 2D). These findings indicate that PNE can ameliorate MC903-induced AD-like symptoms in mice.

### 3.3 PNE ameliorates cytokine and chemokine levels in a mouse model of AD induced by MC903

**FIGURE 3:**
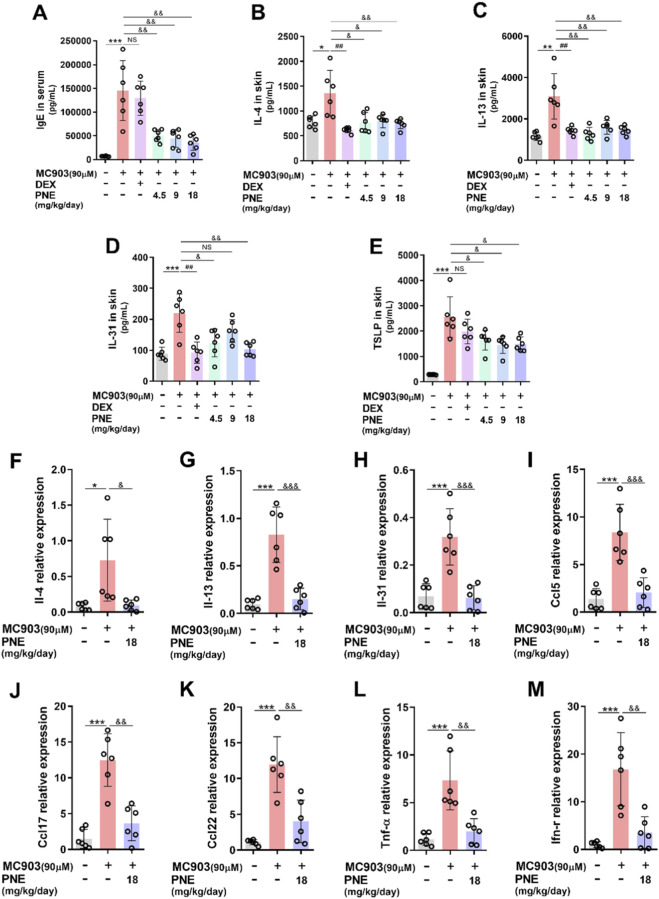
Levels of serum IgE and inflammation-related cytokines/chemokines in skin samples. **(A)** Serum IgE concentrations were quantified by ELISA. **(B–E)** ELISA was used to determine the tissue levels of IL-4, IL-13, IL-31, and TSLP in skin samples. **(F–M)** The expression levels of *Il-4*, *Il-13*, *Il-31*, *Ccl5*, *Ccl17*, *Ccl22*, *Tnf-α*, and *Ifn-γ* in skin tissue were measured by qPCR. Values are expressed as mean ± SD (n = 6). Statistical significance was assessed as follows: comparisons between the MC903 and control group are indicated by *P < 0.05, **P < 0.01, ***P < 0.001; between the DEX and MC903 group by #*P* < 0.05, ##*P* < 0.01, ###*P* < 0.001; and between the PNE and MC903 group by &*P* < 0.05, &&*P* < 0.01, &&&*P* < 0.001.

The elevation of IgE levels indicates that Th2 cytokines predominate in the microenvironment surrounding B cells. In contrast to the MC903-treated group, PNE markedly reduced serum IgE concentrations in mice (Figure 3A). Moreover, AD is characterized by a predominant Th2-type immune response, thus we investigated how PNE modulates the expression of key Th2-associated cytokines (IL-4, IL-13, IL-31, and TSLP) at the site of skin inflammation. As expected, topical application of MC903 triggered a robust upregulation of all four cytokines in the dorsal skin. In contrast, treatment with PNE significantly attenuated their production, albeit to varying degrees (Figures 3B–E). Additionally, administration of PNE (18 mg/kg) decreased the mRNA levels of *Il-4*, *Il-13*, and *Il-31*, which had been elevated by induction of atopic skin lesions (Figures 3F–H). Subsequently, we measured the levels of chemokines (*Ccl5*, *Ccl17*, and *Ccl22*) in MC903-induced AD in mice, which are recognized to recruit Th2 cells capable of secreting Th2 cytokines. As a result, the levels of *Ccl5*, *Ccl17* and *Ccl22* were markedly elevated in MC903-induced AD, whereas PNE significantly reduced these levels (Figures 3I–K). Furthermore, PNE administration decreased the mRNA levels of *Tnf-α* and *Ifn-γ*, which had been elevated by the induction of atopic skin lesions (Figures 3L,M).

### 3.4 MC903-induced upregulation of phosphatidylcholine metabolism in skin tissue was ameliorated by PNE

**FIGURE 4:**
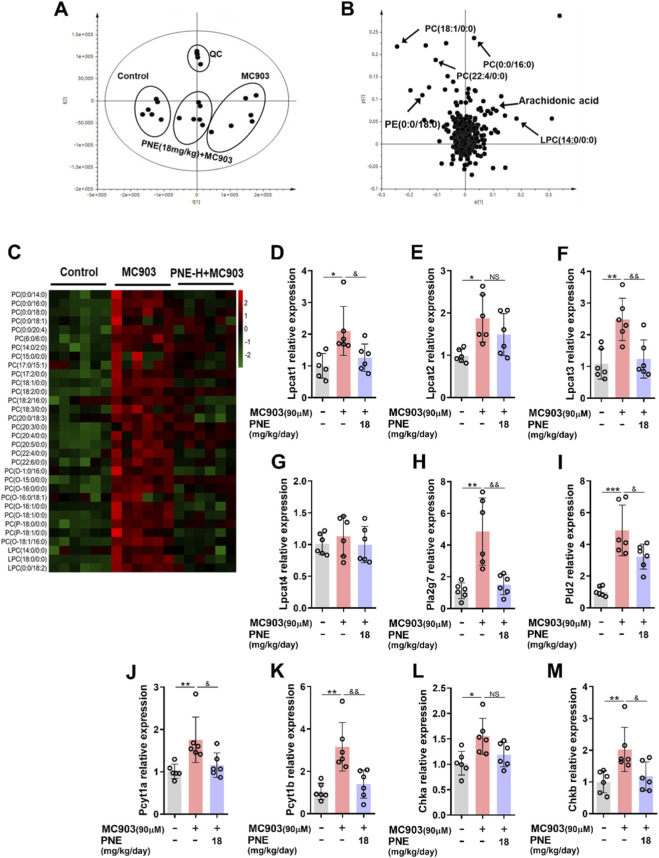
PNE affected the levels of LPCs in the skin tissue of MC903-induced mice. **(A)** OPLS-DA plot of the three groups. **(B)** Loading plot derived from UPLC-MS/MS data of skin tissue metabolites. **(C)** Heatmap of differentially altered LPCs. **(D–H)** Expression levels of genes involved in LPC synthesis and metabolism. **(I–M)** Expression levels of genes involved in PC synthesis and metabolism. Values are expressed as mean ± SD (n = 6). Statistical significance was assessed as follows: comparisons between the MC903 and control group are indicated by *P < 0.05, **P < 0.01, ***P < 0.001; and between the PNE and MC903 group by &*P* < 0.05, &&*P* < 0.01, &&&*P* < 0.001.

In this study, the control group, the MC903 group, and the MC903-treated + PNE-high (18 mg/kg) group were clearly separated on the OPLS-DA score plot (R2X = 0.705, R2Y = 0.98, Q2 = 0.826) (Figure 4A). The OPLS-DA model’s loading scatter plot facilitated the identification of metabolites that contributed most to discriminating between the three groups (Figure 4B). As shown in the heatmap (Figure 4C), PC and its metabolite (LPC) were identified as the most significantly altered lipid species within the phosphatidylcholine family. The mRNA expression levels of key enzymes associated with LPC synthesis and metabolism, including lysophosphatidylcholine acyltransferases 1–4 (*Lpcat1–4*), phosphate cytidylyltransferase 1a and b (*Pcyt1a* and *Pcyt1b*), and choline kinase A and B (*ChkA/B*) were quantified in skin tissue. MC903 treatment markedly upregulated the transcript levels of *Lpcat1*, *Lpcat2*, *Lpcat3*, *Pcyt1a*, *Pcyt1b*, *Chka*, and *Chkb*; however, administration of PNE effectively suppressed this upregulation, except for *Lpcat2* and *Chka* (Figures 4D–M).

### 3.5 MC903-induced upregulation of phosphatidylethanolamine metabolism in skin tissue was alleviated by PNE

**FIGURE 5:**
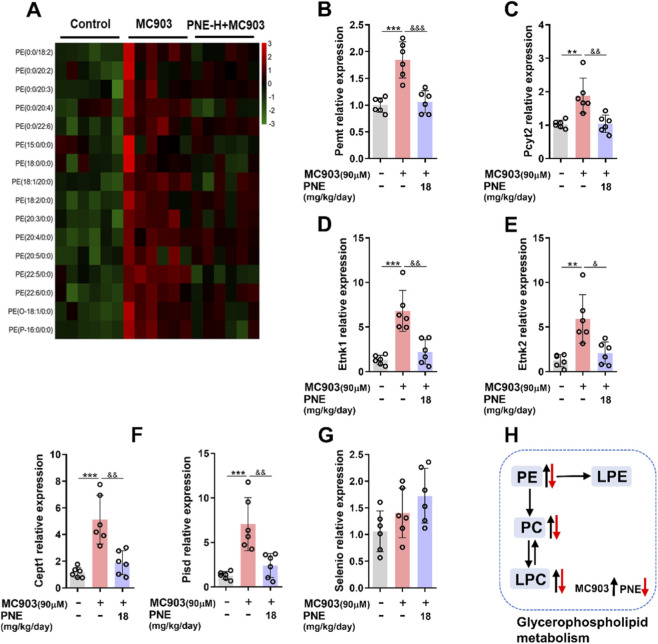
PNE affected the PEs levels in the skin tissue of MC903-induced mice. **(A)** Heatmap analysis of the changed PE. **(B–G)** Gene expression profiles associated with PEs biosynthesis and catabolism. Values are expressed as mean ± SD (n = 6). Statistical significance was assessed as follows: comparisons between the MC903 and control group are indicated by *P < 0.05, **P < 0.01, ***P < 0.001; and between the PNE and MC903 group by &*P* < 0.05, &&*P* < 0.01, &&&*P* < 0.001. **(H)** Schematic illustration of the mechanisms by which PNE modulates Glycerophospholipid metabolism in MC903-induced mice.

Phosphatidylethanolamine (PE) is important for maintaining cell membrane stability as well as mitochondrial morphology and function. The biosynthesis of PE involves the participation of multiple key enzymes, including Phosphatidylethanolamine N-methyltransferase (*Pemt*), Choline/ethanolamine phosphotransferase 1 (*Cept1*), Phosphatidylserine decarboxylase (*Pisd*), Phosphate cytidylyltransferase 2, ethanolamine (*Pcyt2*), Ethanolamine kinase 1/2 (*Etnk1/2*), and Selenoprotein I (*Selenoi*). As illustrated in the heatmap (Figure 5A), MC903 treatment led to a marked upregulation of these PEs relative to the control group; however, administration of PNE effectively attenuated this upregulation. Expression of *Pemt*, *Cept1*, *Pisd*, *Pcyt2*, *Etnk1*, and *Etnk2* mRNAs was markedly upregulated by MC903, and these elevations were reversed by PNE, with the exception of *Selenoi* mRNAs. (Figures 5B–G).

### 3.6 MC903-induced upregulation of arachidonic acid metabolism in skin tissue was ameliorated by PNE

**FIGURE 6:**
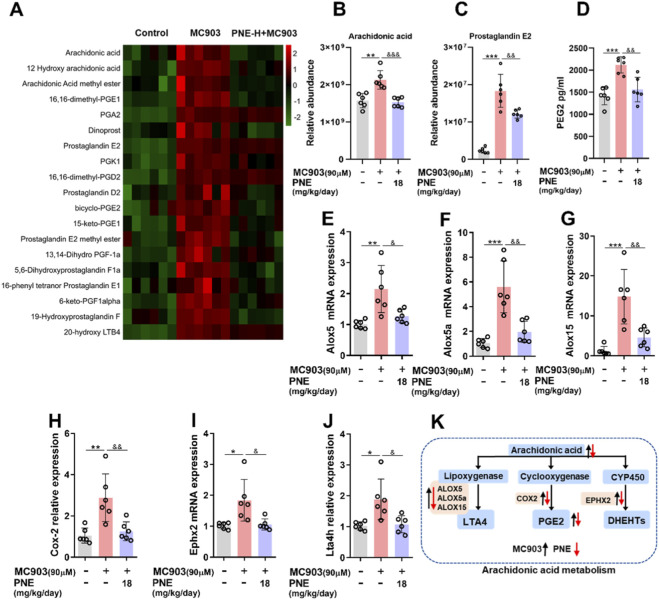
PNE affected the arachidonic acid (AA) metabolism in the skin tissue of MC903-induced mice. **(A)** Heatmap analysis of differentially altered AA-related metabolites. **(B)** PNE decreased arachidonic acid level. **(C)** PNE decreased the PGE_2_ level. **(D)** PGE_2_ levels in skin tissue samples were quantified using an ELISA. **(E–J)** Expression of AA metabolism-related genes. **(K)** Schematic illustration of the mechanisms by which PNE alleviates dysregulated AA metabolism in MC903-induced mice. Values are expressed as mean ± SD (n = 6). Statistical significance was assessed as follows: comparisons between the MC903 and control group are indicated by *P < 0.05, **P < 0.01, ***P < 0.001; and between the PNE and MC903 group by &*P* < 0.05, &&*P* < 0.01, &&&*P* < 0.001.

Arachidonic acid (AA) serves as a key precursor for several enzymatic pathways—the cyclooxygenase, lipoxygenase, and cytochrome P450 systems—yielding a diverse array of biologically active lipid signaling molecules. As shown in the heatmap (Figure 6A), the metabolic profiling analyses significant enrichment of the AA metabolic pathway, within the MC903-induced group, the levels of these relative metabolites exhibited a significant increase compared to the control group, whereas PNE suppressed this elevation. While PGE2 expression levels was increased in the MC903-induced group, whereas PNE suppressed this elevation (Figures 6C,D). The mRNA expression of enzymes responsible for AA metabolism including arachidonate 5-lipoxygenase (*Alox5*), arachidonate 5-lipoxygenase a (*Alox5a*), arachidonate 15-lipoxygenase (*Alox15*), cytochrome c oxidase subunit II (*Cox2*), epoxide hydrolase 2 (*Ephx2*), and leukotriene A4 hydrolase (*Lta4 h*) were measured in skin tissue. MC903 significantly upregulated the mRNA levels of *Alox5*, *Alox5a*, *Alox15*, *Cox2*, *Ephx2*, and *Lta4 h*, whereas PNE treatment effectively counteracted these increases (Figures 6E–J).

### 3.7 PNE regulates the cGAS–STING pathway in MC903-induced AD in mice

**FIGURE 7:**
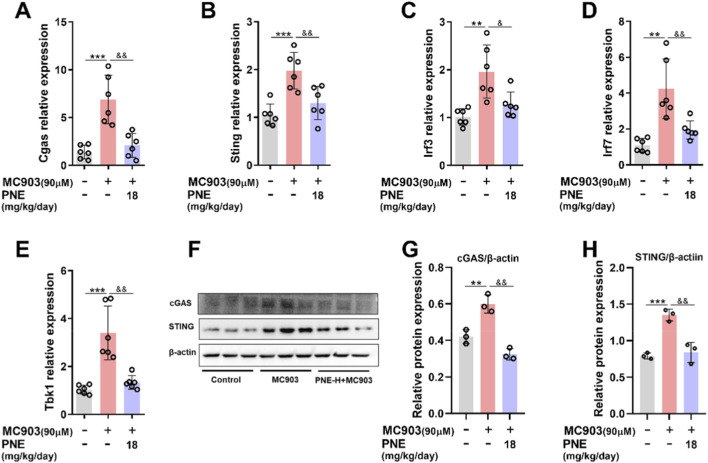
Effect of PNE on the cGAS–STING pathway in MC903-induced mice. **(A–E)** Relative mRNA levels of key genes in the cGAS–STING pathway (*Cgas*, *Sting*, *Irf3*, *Irf7*, and *Tbk1*) were measured by qPCR (n = 4). **(F–H)** The relative expression levels of cGAS and STING proteins were determined by Western blotting (n = 3). Data are represented as mean ± SD. Statistical significance was assessed as follows: comparisons between the MC903 and control group are indicated by *P < 0.05, **P < 0.01, ***P < 0.001; and between the PNE and MC903 group by &*P* < 0.05, &&*P* < 0.01, &&&*P* < 0.001.

To evaluate the potential inhibitory effect of PNE on the cGAS–STING pathway in MC903-induced AD in mice, mRNA levels of *Cgas*, *Sting*, *Irf3*, *Irf7*, and *Tbk1* were analyzed by qPCR. PNE markedly suppressed the MC903-stimulated increase in *Cgas*, *Sting*, *Irf3*, *Irf7*, and *Tbk1* mRNA levels (Figures 7A–E). Additionally, we confirmed that the cGAS and STING protein expression levels was inhibited by PNE treatment (Figures 7F–H). These results indicate that PNE may inhibit the cGAS–STING pathway in MC903-stimulated mice.

### 3.8 PNE reduces the levels of chemokines in TNF-α/IFN-γ-stimulated HaCaT cells

**FIGURE 8:**
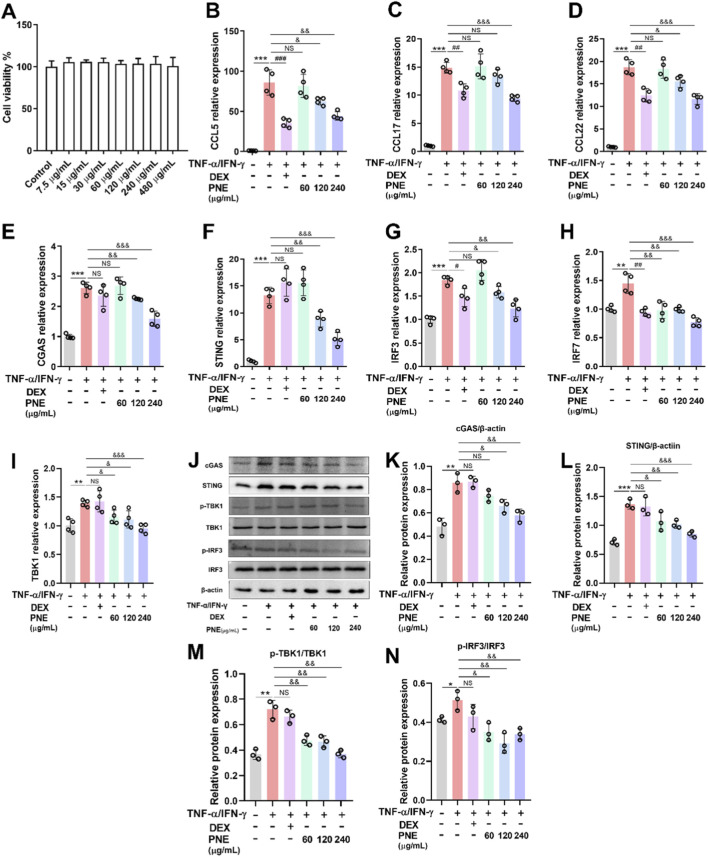
Effect of PNE on the cGAS–STING pathway in TNF-α/IFN-γ co-stimulated HaCaT cells. **(A)** The CCK-8 assay was used to assess the viability of HaCaT cells after 24-h exposure to different concentrations of PNE. **(B–D)** Effects of PNE on the mRNA expression levels of *CCL5*, *CCL17*, and *CCL22* in HaCaT cells. **(E–I)** The relative mRNA levels of key genes in the cGAS–STING pathway (*cGAS*, *STING*, *IRF3*, *IRF7*, and *TBK1*) were measured by qPCR (n = 4). **(J–N)** The relative expression levels of cGAS, STING, p-TBK1 and p-IRF3 proteins were determined by Western blotting (n = 3). Values are expressed as mean ± SD. Statistical significance was assessed as follows: comparisons between the TNF-α/IFN-γ group and the control group are indicated by *P < 0.05, **P < 0.01, ***P < 0.001; between the DEX and TNF-α/IFN-γ group by #*P* < 0.05, ##*P* < 0.01, ###*P* < 0.001; and between the PNE and TNF-α/IFN-γ group by &*P* < 0.05, &&*P* < 0.01, &&&*P* < 0.001.

Keratinocytes are the most abundant cell type in the epidermis and serve as key mediators in the development and progression of inflammatory skin disorders (Cai et al., 2025). Upon activation, these cells secrete a range of chemokines, some of which recruit immune cells capable of producing Th2 cytokines to the site of inflammation, thereby driving localized Th2-mediated immune responses. Thus, we used a well-established cellular model of AD in which HaCaT cells were co-stimulated with TNF-α and IFN-γ to investigate how PNE modulates the secretion of Th2-associated chemokines. The CCK-8 assay showed that PNE did not affect cell viability at concentrations up to 480 μg/mL for 24 h (Figure 8A). HaCaT cells were treated with 60, 120, and 240 μg/mL PNE. TNF-α/IFN-γ co-treatment markedly increased *CCL5*, *CCL17*, and *CCL22* mRNA levels. PNE treatment significantly inhibited the mRNA expression of these chemokines in a dose-dependent manner (Figures 8B–D).

### 3.9 PNE regulates the cGAS–STING pathway in HaCaT cells stimulated with TNF-α/IFN-γ

To evaluate whether PNE inhibits the activation of the cGAS–STING pathway, mRNA levels of *cGAS*, *STING*, *IRF3*, *IRF7*, and *TBK1* were determined by qPCR. PNE significantly inhibited the TNF-α/IFN-γ stimulated upregulation of *cGAS*, *STING*, *IRF3*, *IRF7*, and *TBK1* mRNA levels (Figures 8E–I). Additionally, we confirmed that the cGAS, STING, p-TBK1 and p-IRF3 protein expression levels were inhibited by PNE treatment (Figures 8J–N). The results indicate that PNE may suppress the cGAS–STING pathway.

**FIGURE 9:**
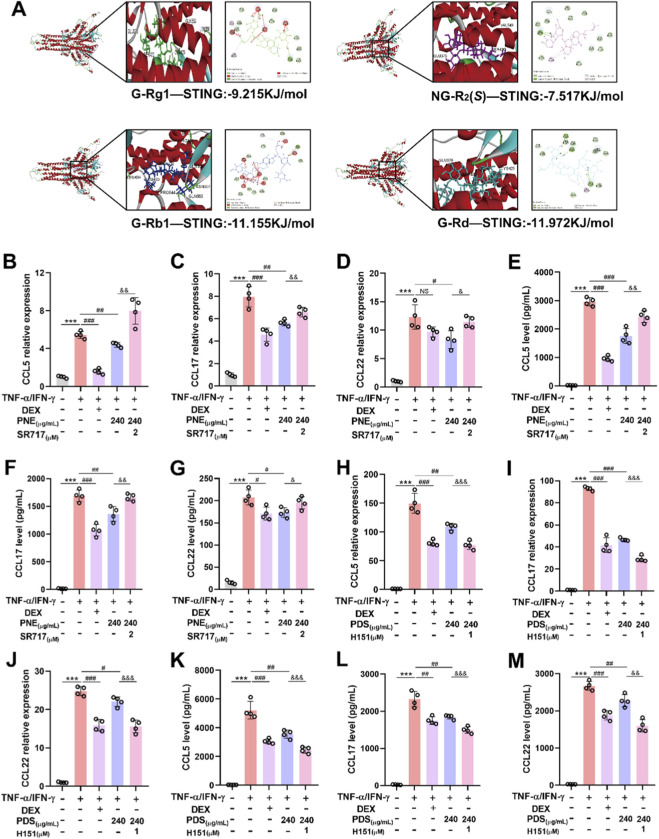
The role of PNE was dependent on the cGAS–STING pathway. **(A)** Molecular docking modeling of G-Rg_1_, NG-R_2_(*S*), G-Rb_1_, and G-Rd with the STING protein. **(B–G)** The protective effect of PNE was attenuated following co-treatment with SR717 (a STING agonist), as measured levels of the inflammatory chemokines (*CCL5*, *CCL17*, and *CCL22*), n = 4. **(H–M)** The protective effect of PNE was enhanced following co-treatment with H151 (a STING inhibitor), as measured levels of the inflammatory chemokines (*CCL5*, *CCL17*, and *CCL22*), n = 4. Values are expressed as mean ± SD. Statistical significance was assessed as follows: comparisons between the TNF-α/IFN-γ group and the control group are indicated by *P < 0.05, **P < 0.01, ***P < 0.001; between the DEX or PNE and TNF-α/IFN-γ group by #*P* < 0.05, ##*P* < 0.01, ###*P* < 0.001; between the SR717 or H151 group and PNE group by &*P* < 0.05, &&*P* < 0.01, &&&*P* < 0.001.

To further confirm whether PNE inhibits the secretion of Th2-associated chemokines by suppressing the cGAS–STING pathway, we performed molecular docking experiments to predict the interactions between the STING protein and the compounds G-Rg_1_, NG-R_2_(*S*), G-Rb_1_, and G-Rd (Figure 9A). The calculated binding affinities of G-Rb_1_, G-Rd, NG-R_2_(*S*), and G-Rg_1_ at the specific sites were −11.155, −11.972, −7.517, and −9.215 kcal/mol, respectively. These binding energies indicate that all four compounds form stable intermolecular interactions with the STING protein. Treatment with the STING agonist SR-717 (2 µΜ) reversed the suppressive effect of PNE on the mRNA expression of *CCL5*, *CCL17*, and *CCL22*, as determined by qPCR (Figures 9B–D). In parallel, TNF-α/IFN-γ stimulation markedly increased the secretion of CCL5, CCL17, and CCL22 in HaCaT cells. PNE effectively inhibited the release of these chemokines, and this inhibitory effect was abolished upon co-treatment with the SR-717, as confirmed by ELISA (Figures 9E–G). Treatment with the STING inhibitor H151 (1 µΜ) enhanced the suppressive effect of PNE on the mRNA expression of *CCL5*, *CCL17*, and *CCL22*, as determined by qPCR (Figures 9H–J). Moreover, PNE effectively inhibited the secretion of these chemokines, and this inhibitory effect was further enhanced upon co-treatment with H151, as confirmed by ELISA (Figures 9K–M). Overall, these results suggest that PNE may suppress the secretion of Th2-associated chemokines by inhibiting the cGAS–STING pathway.

### 3.10 The major components of PNE reduce the levels of chemokines in TNF-α/IFN-γ-stimulated HaCaT cells

**FIGURE 10:**
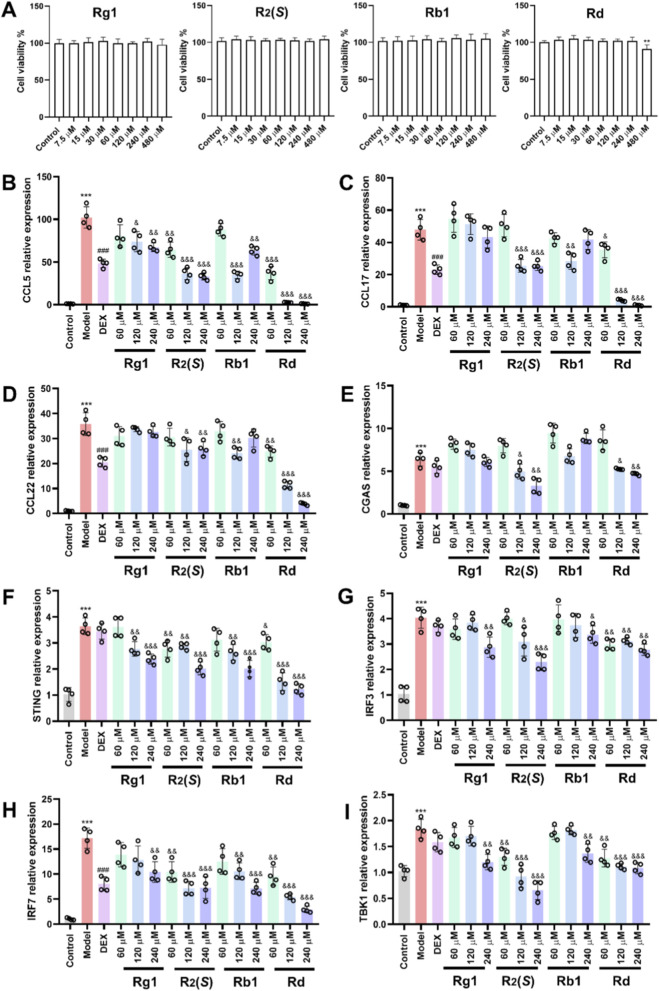
The major components of PNE reduce the levels of chemokines in TNF-α/IFN-γ-stimulated HaCaT cells. **(A)** The CCK-8 assay was used to assess the viability of HaCaT cells after 24-h exposure to different concentrations of G-Rg_1_, NG-R_2_(*S*), G-Rb_1_, and G-Rd. **(B–D)** Effects of G-Rg_1_, NG-R_2_(*S*), G-Rb_1_, and G-Rd on the mRNA expression levels of *CCL5*, *CCL17*, and *CCL22* in HaCaT cells. **(E–I)** The relative mRNA levels of key genes in the cGAS–STING pathway (*cGAS*, *STING*, *IRF3*, *IRF7*, and *TBK1*) were measured by qPCR (n = 4). Values are expressed as mean ± SD. Statistical significance was assessed as follows: comparisons between the TNF-α/IFN-γ group and the control group are indicated by *P < 0.05, **P < 0.01, ***P < 0.001; between the DEX and TNF-α/IFN-γ group by #*P* < 0.05, ##*P* < 0.01, ###*P* < 0.001; and between the Rg_1_, R_2_(*S*), Rb_1_, Rd and TNF-α/IFN-γ group by &*P* < 0.05, &&*P* < 0.01, &&&*P* < 0.001.

The cytotoxicity of the major components of PNE was evaluated by measuring cell viability in HaCaT cells. The cytotoxic effects of G-Rg1, NG-R_2_(*S*), G-Rb1, and G-Rd in HaCaT cells are shown in Figure 10A. Subsequently, the effects of G-Rg1, NG-R_2_(*S*), G-Rb1, and G-Rd on the mRNA expression levels of *CCL5*, *CCL17*, and *CCL22* were assessed in HaCaT cells (Figures 10B–D). By contrast, NG-R_2_(*S*) and G-Rd significantly suppressed the mRNA expression of *CCL5*, *CCL17*, and *CCL22* in a dose-dependent manner. Next, to determine whether G-Rg1, NG-R_2_(*S*), G-Rb1, and G-Rd inhibit activation of the cGAS–STING pathway, we quantified the mRNA levels of *cGAS*, *STING*, *IRF3*, *IRF7*, and *TBK1* by qPCR. Interestingly, NG-R_2_(*S*) and G-Rd significantly inhibited the TNF-α/IFN-γ–induced upregulation of *cGAS*, *STING*, *IRF3*, *IRF7*, and *TBK1* mRNA levels (Figures 10E–I). These results suggest that NG-R_2_(*S*) and G-Rd may suppress the expression of inflammatory chemokines by modulating the cGAS–STING pathway.

## 4 Discussion

*Panax notoginseng* (Burkill) F. H. Chen is a traditional medicinal plant native to southwestern China. Today, it is widely used in global pharmaceutical formulations, dietary supplements, and cosmetic products, contributing substantially to a multi-billion-dollar industry. Experimental evidence has confirmed that saponins extracted from *P. notoginseng* possess potent anti-inflammatory activity and modulate immune function (Wang et al., 2025). Nevertheless, the precise molecular mechanisms underlying the effects of *P. notoginseng* extract (PNE) against AD remain to be elucidated. In this study, we found that PNE may alleviate skin inflammation in AD by inhibiting the cGAS–STING pathway in both mouse and cellular models. Additionally, using metabolomics techniques, we revealed that PNE restores dysregulated skin lipid metabolism in AD.

AD is an inflammatory skin condition primarily driven by type 2 immune responses (Zheng et al., 2021). Central to this process are the Th2-derived cytokines IL-4 and IL-13, which signal through the shared receptor subunit IL-4Rα—a subunit that is widely expressed across multiple cell types, notably B lymphocytes, which regulate IgE production (Pelaia et al., 2022). This signaling axis critically amplifies type 2–mediated cutaneous inflammation. To investigate AD pathophysiology and evaluate therapeutic interventions, several experimentally induced murine models have been established, including those triggered by haptens, whole antigens, or the vitamin D_3_ analog MC903; each model offers unique experimental advantages while also presenting specific methodological limitations. A widely adopted murine model of AD is induced by cutaneous administration of MC903, which triggers a Th2-polarized, AD-mimicking inflammatory response dependent on keratinocyte-derived TSLP. In our study, the MC903-induced AD mouse model was selected based on practical considerations, including model development time and its strong relevance to the core pathophysiological features of AD. In the AD mouse model, PNE exhibited significant anti-AD activity (Figure 2). It markedly reduced serum IgE concentrations (Figure 3A) and simultaneously downregulated key Th2 cytokines (IL-4, IL-13, IL-31, and TSLP) (Figures 3B–E), which likely contributed to the improvement of cutaneous pathology. Notably, localized mast cell accumulation in lesional skin can trigger adjacent keratinocytes to secrete TSLP, a pivotal cytokine that drives Th2 immune polarization and plays a central role in AD (Redhu et al., 2022). Our findings revealed that localized administration of PNE resulted in reduced TSLP levels within the skin tissue.

Th2 cytokines are released by Th2 cells, ILC2 s, and basophils (Ogulur et al., 2025). Elevated local levels of Th2 cytokines suggest the potential recruitment of one or more of these immune cell populations (Kitahata et al., 2022), which can be recruited to inflamed sites by specific chemokines (CCL5, CCL17, CCL22, and TSLP) (Zhang et al., 2015). Thus, we also assessed how PNE affected the concentrations of these chemokines in AD mouse models. The MC903 treatment led to elevated mRNA expression levels of *Ccl5*, *Ccl17*, and *Ccl22* at the lesion site, whereas PNE potently suppressed their expression (Figures 3I–K). Keratinocytes actively contribute to inflammatory responses in the skin by releasing various chemokines (Giustizieri et al., 2001). As key components of the innate immune barrier, keratinocytes are capable of secreting multiple chemokines associated with Th2-type immune responses (Chieosilapatham et al., 2021), including CCL5, CCL17, CCL22, and TSLP (Mali and Bautista, 2021). The observation that topical application of PNE significantly reduced the expression of Th2-type cytokines and chemokines at the affected skin site suggests that PNE’s effect in AD may stem from its ability to dampen keratinocyte hyperactivation.

LC–MS-based metabolomics has been used to explore disease pathogenesis, support timely clinical diagnosis, and monitor therapeutic efficacy in response to pharmacological interventions (Tang et al., 2023). This approach is particularly valuable for investigating inflammatory skin conditions, such as AD and psoriasis, as it facilitates the identification of changes in endogenous metabolites (Schjødt et al., 2020). Moreover, several differentially expressed metabolites, such as prostaglandin J2, S-adenosylmethionine, and 3-hydroxybutyrate, exert significant regulatory effects on anti-inflammatory signaling molecules, thereby contributes to the suppression of inflammatory diseases development (Rodrigues et al., 2021; Li et al., 2021). Therefore, changes in metabolites associated with inflammatory processes indicate that topical application of PNE may help mitigate AD. Many studies indicate that dysregulation of AA metabolism (Huang, R. et al., 2025), glycerophospholipid metabolism (Kaczmarski et al., 2013), and pyrimidine metabolism (Tse-Hung et al., 2018) can initiate and exacerbate inflammatory responses. Our findings indicate that dysregulation of phosphatidylcholine and phosphatidylethanolamine metabolism, along with aberrant signaling in the AA pathway, plays a critical role in AD progression. Notably, PNE-mediated modulation of these metabolic pathways represents a potential therapeutic strategy for managing and preventing allergic inflammation.

The composition of lipids in the stratum corneum plays a critical role in maintaining the skin’s protective barrier; disruptions in this composition are commonly observed in inflammatory dermatoses (Liu, F. et al., 2020). Glycerophospholipids, particularly PC, maintain the structural integrity of cellular membranes and support essential physiological processes. Deviations from their physiological concentrations can exacerbate inflammatory responses and accelerate the progression of pathological conditions (Hishikawa et al., 2014; Tan et al., 2020). LPC, a bioactive lipid generated through the hydrolysis of PC by phospholipase A_2_ (PLA_2_), promotes lymphocyte and macrophage infiltration and stimulates the release of pro-inflammatory cytokines (Liu et al., 2021). Collectively, these effects drive the development and progression of psoriasis (Liu, P. et al., 2020). In our study, the levels of PCs and LPCs were markedly downregulated following PNE administration, suggesting that PNE exerts anti-AD effects by modulating phosphatidylcholine metabolism. TNF-α upregulates the expression of lysophosphatidylcholine acyltransferases two and 4 (LPCAT2/4), enzymes that catalyze the conversion of LPC to PC, thereby reducing LPC levels in nonalcoholic steatohepatitis (Zhang et al., 2019). The observed elevation in LPC levels, coupled with the upregulated mRNA expression of LPCAT2/4, suggests that this increase is attributable to altered expression of genes involved in LPC biosynthesis (Ishino et al., 2024). This change was reversed by PNE treatment, thereby demonstrating the moderating effect of PNE. Consistent with these observations, the expression of *Tnf-α* mRNA was increased in mouse skin tissue following MC903 treatment, and PNE treatment attenuated this increase in MC903-induced AD.

Phosphatidylethanolamine (PE), a key phospholipid component of cellular membranes, is particularly enriched in mitochondrial membranes (Wang et al., 2023). It is essential for maintaining membrane integrity, shaping mitochondrial architecture, and supporting proper mitochondrial function (Zhao et al., 2024). The phosphatidylserine decarboxylation pathway mainly occurs in mitochondria, where phosphatidylserine (PS) is transported to the inner mitochondrial membrane via the intermembrane space protein SLMO2 and then decarboxylated by phosphatidylserine decarboxylase (PISD) to form PE (Zhao et al., 2024). The final step of PE synthesis via the CDP-ethanolamine pathway is catalyzed by choline/ethanolamine phosphotransferase 1 (CEPT1). (Horibata et al., 2020). The expression of *Pemt*, *Cept1*, *Pisd*, *Pcyt2*, *Etnk1*, and *Etnk2* mRNAs was significantly downregulated following PNE administration, suggesting that PNE exerts anti-AD effects by modulating phosphatidylethanolamine metabolism in this study.

The metabolic cascade of AA represents a pivotal regulatory network that orchestrates diverse physiological and pathological processes. As a polyunsaturated fatty acid enriched in the phospholipid bilayer of cell membranes, AA is a bioactive precursor. The process, which is predominantly mediated by enzymes of the phospholipase A_2_ (PLA_2_) family, functions as the rate-limiting step that initiates downstream metabolic branching and is strictly regulated by the cellular microenvironment, the nature of the stimulus, and cell-type-specific receptor signaling. The COX pathway converts AA to prostaglandins (PGs) and thromboxanes (TXs)—a process with well-characterized roles in inflammation, vascular tone, and tissue repair. In our study, the upregulated expression of *Cox-2* mRNA and the accumulation of PGE_2_ were significantly reduced after PNE administration. The LOX pathway, by contrast, generates hydroperoxyeicosatetraenoic acids (HPETEs) and their downstream metabolites, including leukotrienes (LTs) and lipoxins (LXs), with ALOX5 and ALOX15 isoforms mediating cell-type-specific metabolic profiles. LTB_4_ acts as a potent chemoattractant for neutrophils and macrophages, amplifying inflammatory cell infiltration in acute injury and inflammatory diseases. Gene expression levels of several key enzymes involved in AA metabolic pathways (*Alox5*, *Alox5a*, *Alox15*, *Cox2*, *Ephx2*, and *Lta4* *h*) were quantified in skin tissue. The expression of these mRNAs was markedly upregulated by MC903, and these increases were reversed by PNE, suggesting that PNE exerts anti-AD effects by modulating AA metabolism.

**FIGURE 11:**
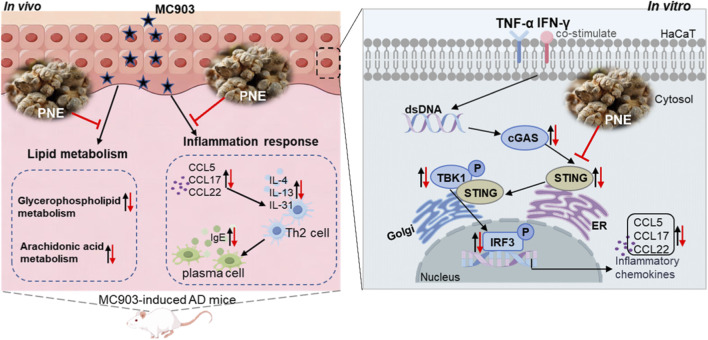
Schematic representation of the effects of PNE on MC903-induced AD in mice and on TNF-α/IFN-γ-activated HaCaT cells.

To investigate the molecular mechanisms underlying PNE’s effects against AD, we used immortalized human HaCaT cells. Stimulation with TNF-α and IFN-γ induced robust secretion of Th2-associated chemokines (CCL5, CCL17, and CCL22) from HaCaT cells. In contrast, PNE effectively suppressed their secretion in a dose-dependent manner (Figures 8B–D). Research indicates that abnormal lipid metabolism induces the release of mitochondrial DNA and activates the cGAS–STING pathway (He et al., 2025). Nanovesicles extracted from *Portulaca oleracea* L., delivered transdermally via microneedles, alleviate AD by reprogramming macrophage polarization toward the anti-inflammatory M2 phenotype and suppressing both the NF-κB and STING signaling pathways (Long et al., 2025). The cGAS–STING pathway serves as a pivotal mediator of pathogenic processes in keratinocytes upon co-stimulation with TNF-α and IFN-γ (Yun et al., 2025). Thus, pharmacological inhibition of this pathway may suppress chemokine secretion by activated keratinocytes. Consistently, PNE treatment significantly downregulated the mRNA and protein expression levels of both cGAS and STING, as demonstrated in both *in vitro* and *in vivo* experiments. Additionally, we confirmed that PNE treatment inhibited the protein expression levels of p-TBK1 and p-IRF3 in HaCaT cells. We used HPLC analysis to confirm that the major constituents of PNE are G-Rg1, NG-R_2_(*S*), G-Rb1, and G-Rd. Molecular docking studies predicted that these compounds exhibit low binding energies toward the STING protein. Moreover, pre-treatment with the STING agonist SR-717 significantly reversed the inhibitory effect of PNE on chemokine secretion in HaCaT cells. Pre-treatment with the STING inhibitor H151 significantly enhanced the inhibitory effect of PNE on chemokine secretion in HaCaT cells. Anti-AD effects of G-Rg1, NG-R_2_(*S*), G-Rb1, and G-Rd were assessed in HaCaT cells. By contrast, NG-R_2_(*S*) and G-Rd significantly suppressed the mRNA expression of *CCL5*, *CCL17*, *CCL22*, *cGAS*, *STING*, *IRF3*, *IRF7*, and *TBK1* in a dose-dependent manner (Figure 10). These findings support the hypothesis that the anti-AD effects of PNE may be attributed to NG-R_2_(*S*) and G-Rd. In summary, our study suggests that PNE may exert its effect by suppressing the cGAS–STING pathway, thereby reducing chemokine production by keratinocytes, hindering the recruitment of cells that secrete Th2 cytokines, decreasing local levels of IL-4, IL-13, and IL-31, and ultimately alleviating AD-associated skin lesions (Figure 11).

In China, over 500 approved drugs contain bioactive compounds derived from PN, primarily used for treating trauma, ischemic stroke, and thrombotic disorders (source: Chinese Marketed Drugs Database). Additionally, more than 70 clinical studies support the efficacy of PN-derived extracts and traditional formulae in treating cerebral infarction, stroke rehabilitation, osteoarthritis, and intracerebral hemorrhage (Yang et al., 2024). The PNE is of natural origin and offers safety advantages. Moreover, using the MC903-induced AD model, this study demonstrated for the first time that PNE exhibits significant anti-AD effects. NG-R_2_(*S*) and G-Rd are likely the active components responsible for this activity. These findings provide a foundation for the clinical translation and application of PNE in AD treatment.

Although this study has uncovered important findings, it also has several limitations. These primarily include the following: (a) As key constituents of the extract, G-Rd and NG-R_2_(*S*) require further animal and clinical experimental verification of their anti-AD effects. (b) Our experiments were conducted primarily in cell and animal models, and the translational relevance to human physiology and disease requires further validation using clinical samples. (c) We focused on metabolic enzymes and major metabolites involved in lipid metabolism, while their modulatory roles in AD remain to be elucidated. We recognize the limitations of this study. However, our results are interesting, indicating that PNE holds therapeutic promise for AD and may contribute to elucidating the underlying molecular mechanisms. Subsequent studies should focus on investigating its effects on lipid metabolism in relation to pruritus, and clinical trials of PNE will be conducted.

## 5 Conclusion

This research indicates that PNE may alleviate AD-like skin inflammation by modulating the cGAS–STING pathway. Our findings suggest that PNE could serve as a potential therapeutic option for AD.

## Data Availability

The raw data supporting the conclusions of this article will be made available by the authors without undue reservation. The untargeted metabolomic data used in this publication have been submitted to the EMBL-EBI MetaboLights database with the identifier MTBLS14871. The complete data set can be accessed at https://www.ebi.ac.uk/metabolights/MTBLS14871.
