# Supplementary material for: Panax notoginseng extract alleviates atopic dermatitis-like skin inflammation associated with suppression of the cGAS–STING pathway
Source: Front Pharmacol. 2026 Jul 9;17:1859988. doi: 10.3389/fphar.2026.1859988 (PMC13393094; doi:10.3389/fphar.2026.1859988)
Supplement: Supplementary file 1 [file Supplementaryfile1.pdf]

Supplementary Materials

Panax notoginseng extract alleviates atopic dermatitis-like skin inflammation associated with suppression of the cGAS-STING pathway

Xu Zhu [1,2], Yichun Wang [1,2], Zhongyi Chen [1,2], Ziyan Zhao [1,2], Chunhao Yang [1,2], Peng Xu [1,2], Liping Qu [1,2,3], Feifei Wang [1,2,3,*], Fengkun Xiao [1,2, **]

[1] Yunnan Characteristic Plant Extraction Laboratory Co., Ltd., Kunming, 650106, China

[2] Yunnan Botanee Bio-technology Group Co., Ltd., Kunming, 650106, China

[3] Botanee Research Institute, Shanghai Jiyan Biomedical Development Co., Ltd., Shanghai, 201702, China

* Corresponding author at: Yunnan Characteristic Plant Extraction Laboratory Co., Ltd., Kunming, 650106, China.

** Corresponding author at: Yunnan Characteristic Plant Extraction Laboratory Co., Ltd., Kunming, 650106, China.

E-mail addresses: wangfeifei@botanee.com (F. Wang), xiaofengkun@botanee.com (F. Xiao).

This file includes:

Table S1

Table S2

Table S3

Table S4

Table S1.

The extraction yield and batch-to-batch consistency data for PNE

Batch	Yield	G-Rg1	NG-R2(*S*)	G-Rb1	G-Rd
1	7.84%	13.86%	3.99%	39.79%	7.72%
2	7.57%	14.66%	4.31%	42.01%	8.27%
3	7.63%	14.98%	4.41	43.02%	8.39%

Table S2.

Clinical scoring criteria for atopic dermatitis in MC903-induced mouse models

Symptom \ Score	0	1	2	3
Erythema	No	Light erythema rubrum	Obvious redness	Bright red or deep red in color
Edema/papules	No	Localized dermal edema in certain areas is not easily palpable	There was definite dermal edema in multiple areas	Extensive dermal edema occurred over a wide area
Dryness/desquamation	No	There is mild local desquamation, mainly consisting of fine scales	There are multiple areas of desquamation on the body, and the scales are relatively coarse	There is significant desquamation over most parts of the body, with thick and coarse scales
Lichenification	No	The skin texture is slightly thickened	The skin texture thickens and exhibits a crisscross pattern	The skin texture is significantly thickened and presents a very deep, crisscross pattern

Table S3.

Primer sequences used for real-time PCR in mice

Genes	Sequences		Species
	Forward	Reverse	
Gapdh	5'-CATCACTGCCACCCAGAAGACTG-3'	5'-ATGCCAGTGAGCTTCCCGTTCAG-3'	mouse
Il-4	5'-GGTCTCAACCCCCAGCTAGT-3'	5'-GCCGATGATCTCTCTCAAGTGAT-3'	
Il-13	5'-CCTGGCTCTTGCTTGCCTT-3'	5'-GGTCTTGTGTGATGTTGCTCA-3'	
Il-31	5'-TCAGCAGACGAATCAATACAGC-3'	5'-TCGCTCAACACTTTGACTTTCT-3'	
Ccl5	5'-TTTGCCTACCTCTCCCTCG-3'	5'-CGACTGCAAGATTGGAGCACT-3'	
Ccl17	5'-TACCATGAGGTCACTTCAGATGC-3'	5'-GCACTCTCGGCCTACATTGG-3'	
Ccl22	5'-CTCTGCCATCACGTTTAGTGAA-3'	5'-GACGGTTATCAAAACAACGCC -3'	
Tnf-α	5'-GTCTCAGCCTCTTCTCAT-3'	5'-GAACTTCTCATCCCTTTGG-3'	
Ifn-γ	5'-CTCAAGTGGCATAGATGTG-3'	5'-GAAGGTAGTAATCAGGTGTG-3'	
Cnka	5'-AAAGTGCTCTTGCGGCTCTA-3'	5'-GACCTCTCTGCAAGAATGGC-3'	
Cnkb	5'-GCAGAGGTTCAGAAGGGTGA-3'	5'-CCCCAGAAAAAGTGAGATGC-3'	

Pcyt1a	5'-AGCCCTATGTCAGGGTGACT-3'	5'-GGCATGACCAGAGTGAAACA-3'
Pcyt1b	5'-ATAGAGCACACATGCCCACA-3'	5'-GGCAACGGTCAGTTTTTCAT-3'
Pld2	5'-CAGCTACATCAGCATGACAGC-3'	5'-CTGCCACAGCAGCAAAGTAA-3'
Lpcat1	5'-CACGAGCTGCGACTGAGC-3'	5'-ATGAAAGCAGCGAACAGGAG-3'
Lpcat2	5'-ACCTGTTTCCGATGTCCTGA-3'	5'-CCAGGCCGATCACATACTCT-3'
Lpcat3	5'-AGCCTTAACAAGTTGGCGAC-3'	5'-ATGCCGGTAAAACAGAGCC-3'
Lpcat4	5'-GAGTTACACCTCTCCGGCCT-3'	5'-GGCCAGAGGAGAAAGAGGAC-3'
Pla2g7	5'-ATTTCTTGGAACACCCAGTATTGT-3'	5'-GAACATTCTATTGCTCTTTGCTGA-3'
Cept1	5'-ATGAGTGGGCATCGGTCAAC-3'	5'-GTGGTGTCGGTAACTGAAACAA-3'
Pisd	5'-CATACTGCTCCTGTCCGATCC-3'	5'-TTCCGTTCCCTGTACTTCTCATA-3'
Etnk1	5'-CTGTTCACAGATGGGATCACAA-3'	5'-GCCGTAAATCCTCACCAGAACTA-3'
Etnk2	5'-CGGTGGAACAGGACGACATC-3'	5'-AGGCCAATAGCTTGTTGGTGA-3'
Pcyt2	5'-TGGTGCGATGGCTGCTATG-3'	5'-CCCTTATGCTTGGCAATCTCC-3'
Pemt	5'-ATCACCATTGTGTTCAACCCAC-3'	5'-CCAGGGAATAGCAGGCTAGG-3'
Selenoi	5'-ACTGCTTCCTCTCCTCTT-3'	5'-GCTGCTTCACCACTTGTA-3'
Alox5	5'-GGGCTGTAGCGAGAAGCATC-3'	5'-CACGGTGACATCGTAGGAGT-3'
Alox5ap	5'-GCATGAAAGCAAGGCGCATAA-3'	5'-GGTACGCATCTACGCAGTTCT-3'
Alox15	5'-GGCTCCAACAACGAGGTCTAC-3'	5'-CCCAAGGTATTCTGACACATCC-3'
Lta4h	5'-GAGGTCGCGGATACTTGCTC-3'	5'-TTTTGTGTCCAAAGTCAGGCT-3'
Ephx2	5'-CTTGGTGCGTACCAGACGG-3'	5'-TTCTCAGGTAGATTGGCTCCA-3'
Cgas	5'-TAGATGTCAGTGTGGAGAAG-3'	5'-TCGTCTTAGATTGGTCCTC-3'
Sting	5'-GCCTGGTCATACTACATTG-3'	5'-GCAGCATATCTCGGAATC-3'
Irf3	5'-GAGAGCCGAACGAGGTTCAG-3'	5'-CTTCCAGGTTGACACGTCCG-3'
Irf7	5'-GCGTACCCTGGAAGCATTTC-3'	5'-GCACAGCGGAAGTTGGTCT-3'
Tbk1	5'-GGAGCCGTCCAATGCGTAT-3'	5'-GCCGTTCTCTCGGAGATGATTC-3'

Table S4.

Primer sequences used for real-time PCR in human

Genes	Sequences	Species

	Forward	Reverse	
GAPDH	5′-TCAAGTGGGGCGATGCTGGC-3′	5′-TGCCAGCCCCAGCGTCAAAG-3′	human
CCL5	5'-CCAGCAGTCGTCTTTGTCAC-3'	5'-CTCTGGGTTGGCACACACTT-3'	
CCL17	5'-CCAGGGATGCCATCGTTTTTG-3'	5'-TAGTCCCGGGAGACAGTCAG-3'	
CCL22	5'-ATCGCCTACAGACTGCACTC-3'	5'-GACGGTAACGGACGTAATCAC-3'	
CGAS	5'-GATATAACCCTGGCTTTGG-3'	5'-GTCGTAGTTGCTTCCTAAC-3'	
STING	5'-CAGCAACAGCATCTATGAG-3'	5'-CCACAGTAACCTCTTCCTT-3'	
IRF3	5'-AGAGGCTCGTGATGGTCAAG-3'	5'-AGGTCCACAGTATTCTCCAGG-3'	
IRF7	5'-GCTGGACGTGACCATCATGTA-3'	5'-GGGCCGTATAGGAACGTGC-3'	
TBK1	5'-TGGGTGGAATGAATCATCTACGA-3'	5'-GCTGCACCAAAATCTGTGAGT-3'	
